# Mo-LDH-GO Hybrid Catalysts for Indigo Carmine Advanced Oxidation

**DOI:** 10.3390/ma16083025

**Published:** 2023-04-11

**Authors:** Octavian Dumitru Pavel, Alexandra-Elisabeta Stamate, Rodica Zăvoianu, Anca Cruceanu, Alina Tirsoaga, Ruxandra Bîrjega, Ioana Andreea Brezeștean, Alexandra Ciorîță, Daniela Cristina Culiță, Ana Paula Soares Dias

**Affiliations:** 1 Department of Inorganic Chemistry, Organic Chemistry, Biochemistry and Catalysis, Faculty of Chemistry, University of Bucharest, 4-12 Regina Elisabeta Bd., 030018 Bucharest, Romania; 2 Research Center for Catalysts & Catalytic Processes, Faculty of Chemistry, University of Bucharest, 4-12 Regina Elisabeta Bd., 030018 Bucharest, Romania; 3 National Institute for Lasers, Plasma and Radiation Physics, 409 Atomistilor Street, 077125 Măgurele, Romania; 4 National Institute for Research and Development of Isotopic and Molecular Technologies, 67-103 Donat Street, 400293 Cluj-Napoca, Romania; 5 Electron Microscopy Centre, Faculty of Biology and Geology, Babes-Bolyai University, 44 Republicii Street, 400015 Cluj-Napoca, Romania; 6 Ilie Murgulescu Institute of Physical Chemistry, 202 Splaiul Independentei, 060021 Bucharest, Romania; 7 CERENA, Instituto Superior Técnico, Universidade de Lisboa, 1 Rovisco Pais Av., 1049-001 Lisboa, Portugal

**Keywords:** advanced oxidation process, wastewater treatment, hybrid catalysts, Mo-modified LDH, graphene oxide, indigo carmine

## Abstract

This paper is focused on the utilization of hybrid catalysts obtained from layered double hydroxides containing molybdate as the compensation anion (Mo-LDH) and graphene oxide (GO) in advanced oxidation using environmentally friendly H_2_O_2_ as the oxidation agent for the removal of indigo carmine dye (IC) from wastewaters at 25 °C using 1 wt.% catalyst in the reaction mixture. Five samples of Mo-LDH-GO composites containing 5, 10, 15, 20, and 25 wt% GO labeled as HTMo-xGO (where HT is the abbreviation used for Mg/Al in the brucite type layer of the LDH and x stands for the concentration of GO) have been synthesized by coprecipitation at pH 10 and characterized by XRD, SEM, Raman, and ATR-FTIR spectroscopy, determination of the acid and base sites, and textural analysis by nitrogen adsorption/desorption. The XRD analysis confirmed the layered structure of the HTMo-xGO composites and GO incorporation in all samples has been proved by Raman spectroscopy. The most efficient catalyst was found to be the catalyst that contained 20%wt. GO, which allowed the removal of IC to reach 96.6%. The results of the catalytic tests indicated a strong correlation between catalytic activity and textural properties as well as the basicity of the catalysts.

## 1. Introduction

In the last decade, interest in obtaining new hybrid materials containing layered double hydroxides (LDH) and graphene oxide (GO) has constantly increased [1]. LDH are anionic clays with a 2D lamellar structure composed of positively charged layers containing bivalent and trivalent cations hexacoordinated with hydroxyl groups as in the brucite (Mg(OH)_2_) structure and negatively charged layers containing compensation anions and water molecules. The general formula of LDH is [M^II^_1−x_M^III^_x_(OH)_2_]^x+^[A^n−^]_x/n_·zH_2_O, where M^II^ is a bivalent metal cation such as Mg, Zn, Ni, Co, etc., M^III^ is a trivalent metal cation, (e.g., Al, Ga, Fe, Cr), A^n−^ is a charge compensation anion, which may be either inorganic or organic, and x has a value between 0.2 and 0.4 [2,3]. Meanwhile, GO can be obtained by the oxidation of graphite [4] or by shredding and oxidizing graphene. It has a hexagonal carbon structure and contains different oxygen-based functional groups, such as: hydroxyl (–OH), alkoxy (C–O–C), carbonyl (C=O), and carboxyl (–COOH) [1,5]. GO has a large surface area and a high oxygen content (the carbon/oxygen ratio of GO is in the range 2:1–3:1). In hybrid LDH-GO materials, the positively charged brucite-type layers of the LDH are electrostatically attracted by negatively charged oxygen-containing functional groups from the GO surface. As a result, the surface of the hybrid is larger than that of the LDH alone, the stability of the LDH is increased, and the anionic exchangeability of the LDH is preserved [5].

The first mention of LDH-GO hybrids described the obtaining of bidimensional (2D) nanostructured materials by immobilization of ZnCr-LDH positively charged nanoplates on the surface of negatively charged graphene nanolayers using the self-assembly method. The resulting hybrids, ZnCr-LDH-GO and ZnCr-LDH-RGO (RGO-reduced graphene oxide) were used as active photocatalysts to generate O_2_ under visible light radiation [6]. Since then, many other publications have referred to the use of LDH-GO hybrids containing transition metals (Zn/Fe or Ni/Cr) as photocatalysts for advanced oxidation processes in water treatment for the removal of pharmaceuticals [7,8,9], while the removal of dye pollutants was attempted only for the case of methylene blue [10] using Co/Al. Recently, a ternary LDH structure TiMgAl-LDH combined with GO was used for the photocatalytic reduction of CO_2_ [11]. Due to their exceptional electronic and conduction properties, LDH-GO hybrids have been used as sensors to determine guanine, adenine, papaverine, non-enzymatic sugars, or hydrogen peroxide [12,13,14] as well as energy storage systems [15]. LDH-GO hybrids containing Ni, Fe, Co, and Cu in the LDH structure have also been used as electrocatalysts for different types of processes involved in fuel and energy production [16,17,18,19,20].

In addition to their utilization in photocatalysis and electrocatalysis, LDH-GO hybrids acted as classical catalysts for various processes starting with NO_x_ reduction on Pt-doped MgAl-LDH-GO in 2015 [21], followed by the Ullmann carbon-carbon coupling reaction on CuAl-LDH-GO and CoAl-LDH-GO [22], the one-pot oxidation-Knoevenagel condensation reaction on Ru-MgAl-LDH-GO [23] or CeMgAl-LDH-GO [24] and the degradation of gatifloxacin on CoFeNi-LDH-GO [25].

Before being used in wastewater treatment by advanced oxidation processes, LDH-GO hybrid materials were also utilized as adsorbents for different pollutants. Thus, the first reference related to the use of LDH-GO composites in water treatment published in 2014 describes the removal of cadmium ions and methylene blue dye by adsorption on 3D hybrid aerogels made by cross-linking MgAl-LDH and GO [26]. Recently, the removal of organic dyes with LDH-GO adsorbents was realized using CoZnAl-LDH-GO for methylene blue adsorption [27] and ZnAl-LDH-GO as adsorbent of methyl orange [28]. Hybrid composites MgAl-LDH-GO with polysulfone [29] or polyvinylidene fluoride [30] were used as membranes to remove Cu^2+^ and methylene blue from wastewater by osmosis.

Most of the research on LDH-GO hybrids conducted to date has been limited to the incorporation in the GO matrix of LDH structures with inorganic compensation anions such as Cl^−^, NO_3_^−^, CO_3_^2−^, SO_4_^2−^ and only one mention of a hybrid composite, used as a corrosion inhibitor for carbon steel, related to the incorporation of a molybdate intercalated LDH combined with GO [31]. Mo-modified layered double hydroxides (Mo-LDH) are synthetic anionic clays, which may contain molybdenum either as molybdate (MoO_4_^2−^) or heptamolybdate (Mo_7_O_24_^6−^) compensation anions, depending on the pH value during the synthesis [32,33,34,35]. It has been proved that molybdate-containing LDH are selective catalysts for the oxyfunctionalization of organic substrates due to their ability to generate single molecular oxygen from hydrogen peroxide [32,36,37] following a sequential mechanism involving in the first step the formation of monoperoxomolybdate MoO_3_(O_2_)^2−^, which is further transformed into diperoxomolybdate MoO_2_(O_2_)_2_^2−^, triperoxomolybdate MoO(O_2_)_3_^2−^ and tetraperoxodimolybdate Mo_2_O_3_(O_2_)_4_^2−^ species on the surface of the catalyst [32,38]. Hence, it may be considered that Mo-LDH could also be active in the advanced oxidation process for water treatment when using H_2_O_2_ as a green oxidation agent.

The textile industry is considered one of the most significant pollutant sources of water bodies since around 15% of the dyes are discarded and released in emerging effluents after the dyeing process [39,40]. One of the issues posed by dye effluents is that even when these are found in low concentration, they affect water transparency hindering the evolution of aquatic species and having harmful and sickening effects. Another issue is their intricate degradation when classical water treatments are applied. Advanced oxidation processes are important in wastewater treatment because the scarcity of potable water in some parts of the world requires the treatment of wastewater to make it potable [41,42,43,44]. These procedures can also be used to improve the odor and taste of some drinking water sources whose geological origin causes them to have less desirable features for these parameters [45].

**Figure 1:**
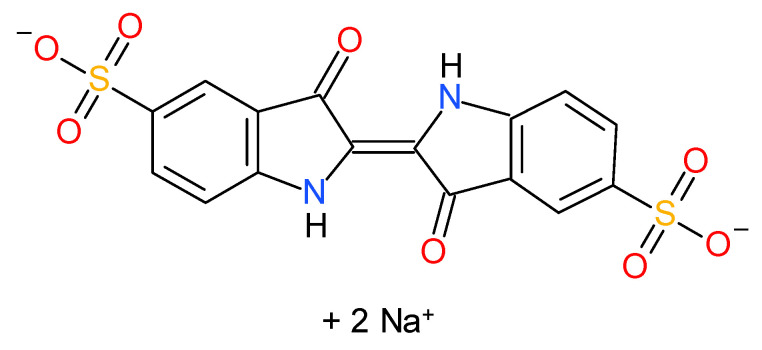
The chemical structure of indigo carmine (IC).

Disodium (2E)-3-oxo-2-(3-oxo-5-sulfonato-2,3-dihydro-1H-indol-2-ylidene)-2,3-dihydro-1H-indole-5-sulfonate also known as indigo carmine (IC), indigotin, or Acid Blue 74 is an indigoid water-soluble dye (Figure 1) that is frequently used in concentrations ranging between 0.21 to 4.5 mmol L^−1^ in the textile industry related to blue-denim fabrics [46]. The dye is also used in small amounts as an additive in some pharmaceutical formulations, or as a staining agent for medical diagnostic purposes [47,48]. It can have toxic effects on humans leading to reproductive, cardiovascular, respiratory, developmental, and neuronal disorders, as well as carcinogenic effects by provoking tumors at the site of application [47,48] or when the doses are higher than 500 mg/kg body weight/day [49].

Until now, IC removal has been attempted by different advanced oxidation processes (AOP) such as: photocatalysis using TiO_2_ [50,51,52], photo-Phenton [52], ultrasonic assisted electrocatalysis on MnO_2_ catalysts using peroxydisulfate as the oxidant [53], electrocatalysis on Ti/IrO_2_-SnO_2_-Sb_2_O_5_ [52] or PbO_2_/Fe [54], and catalysis on hematite-derived nanocomposites using H_2_O_2_ as the oxidant [55]. However, there are several disadvantages to these processes since photocatalysis and electrocatalysis imply higher costs of the equipment required for industrial applications, the use of TiO_2_ as a photocatalyst did not lead to a substantial loss in the total organic carbon content of the water even if the water coloration disappeared [50], while the use of peroxydisulfate as an oxidation agent implies the generation of sulfate as a by-product, which pollutes the water. From both the economic and environmental points of view, the more favorable systems are those based on conventional catalysis using H_2_O_2_ as an oxidation agent.

Considering this state of the art, our contribution is focused on the synthesis, characterization, and catalytic activity testing in oxidative dye removal from wastewater of new hybrid composites based on Mo-containing LDH and graphene oxide with different GO loading. It was assumed that using the co-precipitation method at pH 10, the inclusion of MoO_4_^2−^ compensation anions, which can activate the H_2_O_2_ molecules in the LDH will be favored. Moreover, incorporating GO will enhance the affinity of the resulting solids for the organic dye substrate.

## 2. Materials and Methods

The chemicals necessary for the synthesis of the Mo-LDH phase, e.g., magnesium nitrate hexahydrate Mg(NO_3_)_2_∙6H_2_O, aluminum nitrate nonahydrate Al(NO_3_)_3_∙9H_2_O, sodium molybdate dihydrate Na_2_MoO_4_∙2H_2_O, anhydrous sodium carbonate Na_2_CO_3_, and sodium hydroxide NaOH (pearls), were all of chemical purity grade and were purchased from Merck (Darmstadt, Germany). For the preparation of the GO phase, graphite powder 325 mesh from Sigma–Aldrich (Saint Louis, MO, USA), sodium nitrate NaNO_3_, potassium permanganate KMnO_4_ (chemical purity from Merck), H_2_SO_4_ (98%), hydrochloric acid HCl 37% (from Merck), and hydrogen peroxide H_2_O_2_ 30% (from ChimReactiv, Bucharest, Romania) were utilized.

Indigo carmine (IC) from Sigma–Aldrich (Saint Louis, MO, USA) was used to prepare the simulated dye-contaminated water in the laboratory.

All the aqueous solutions were obtained using distilled water with a conductivity of 2.5–5 μS/cm.

### 2.1. Materials Synthesis

A sample of LDH containing molybdate as the compensation anion was prepared by co-precipitation at pH 10 to serve as a reference. To this aim, two solutions have been prepared using distilled water as solvent: (*i*) solution A containing Mg(NO_3_)_2_∙6H_2_O and Al(NO_3_)_3_∙9H_2_O at a molar ratio Mg/Al = 3 and a concentration of cations (Mg + Al) equal to 0.35 M and (*ii*) solution B containing NaOH (0.76 M) and an amount of Na_2_MoO_4_∙2H_2_O equal to 50 molar percent of the amount of Al(NO_3_)_3_∙9H_2_O dissolved in the solution A (molar ratio Mo/Al = 0.5:1). The molar ratio between NaOH and the sum of cations in solution A was 2.2:1. The precipitation was carried out at room temperature under vigorous stirring (300 rpm) using a TIM854 Titration Manager from Radiometer Analytical (Budapest, Hungary), which allows a constant pH value to be maintained by the addition of the required amounts of the pH-adjusting solution B from an automatic burette. Solution A and solution B were added concomitantly in the precipitation reactor, which already contained 50 mL of distilled water brought to pH 10. After finishing the addition of solutions A and B, another portion of 50 mL distilled water was added, a vertical condenser was fixed on the top of the reactor, and the obtained gel was heated at 70 °C and aged for 18 h. The aged gel was separated by filtration and the resulting cake was thoroughly washed with distilled water to remove the soluble by-product salts. The washing ended when the conductivity of the washing water fell below 100 μS/cm. The cake was dried in an oven with air circulation at 90 °C for 24 h. The dried sample was designated as HTMo.

Five samples of Mo-LDH-GO composites containing 5, 10, 15, 20, and 25 wt% GO labeled as HTMo-xGO (where HT is the abbreviation used for Mg/Al in the brucite type layer of the LDH and x stands for the concentration of GO) were synthesized by co-precipitating the LDH phase in the presence of GO at pH 10, similar to the method applied by us for the synthesis of Ce-LDH-GO hybrids [24]. To this aim, a suspension of GO with a concentration of 4 g/L was prepared using Hummers’ method [4] as it was described in reference [24]. The details of the preparation of the GO suspension are included in Supplementary Materials Paragraph S1.

Identical preparation steps were applied for all the composites, the differences between their synthesis consisting in the amounts of precursor salts used (respecting the molar ratios Mg:Al of 3:1 and Mo/Al of 0.5:1), which varied with the concentration of GO to be included in the resulting solid. Solution A contained the required amounts of Mg and Al nitrates solubilized in a mixture of distilled water and the necessary quantity of GO suspension. Solution B was prepared as described for the synthesis of HTMo, respecting the molar ratio of NaOH/(Mg + Al) equal to 2.2:1 and Mo/Al of 0.5:1. The detailed compositions of the reaction mixtures are presented in Supplementary Materials Table S1. All the procedures related to equipment and pH of the precipitation, aging, washing, and drying of the obtained solids were the same as for the synthesis of HTMo. The obtained samples were labeled HTMo-5GO, HTMo-10GO, HTMo-15GO, HTMo-20GO, and HTMo-25GO.

### 2.2. Materials Characterization

Inductively coupled plasma atomic emission spectroscopy (ICP-AES), was used to determine the metal content of the samples using a Liberty 110 spectrometer from Varian (Palo Alto, CA, USA). To this aim, the samples were first calcined for 8 h in an air flow (10 mL/min) at 500 °C to remove the graphene oxide and then the metals from the remaining ashes were solubilized in ultrapure nitric acid.

The total number of acid sites was determined by pyridine adsorption using the methods described in reference [24]. For the determination of the total number of base sites, a method based on the irreversible adsorption of organic acids as described in reference [34], (e.g., acrylic acid, pKa = 4.2 for the determination of the total number of base sites and phenol pKa = 9.9, for the determination of the number of strong acid sites) was used. Before these determinations, the samples were thoroughly degassed under vacuum at room temperature.

XRD powder patterns were recorded on a Panalytical X’Pert θ/2θ-diffractometer (from Panalytical, Almelo, Netherlands) equipped with an Xcelerator detector using Cu-Kα radiation (40 kV, 40 mA; λ = 1.5418 Å nm). The diffractograms were collected with a step of 0.02°/min and an acquisition time per step of 4 s. Peak positions and profiles were fitted with the Pseudo-Voigt function using the HighScore Plus software package version 2014 (Panalytical). The PDF-4+ database of the International Center of Diffraction Data (ICDD) was used for phase identification. Scherrer’s formula (1) was used to calculate the dimensions of the crystallites:

(1) $$D_{hkl}=\frac{K\cdot \lambda }{\beta_{hkl}\cdot cos\theta_{hkl}}$$

where K is a geometric factor (e.g., 0.9), λ is the wavelength of the incident X-ray, $\beta_{hkl}$ is the width at half-intensity of the hkl reflection, and $\theta_{hkl}$ is the Bragg angle of the same reflection.

Attenuated total reflectance Fourier transformed infrared (ATR-FTIR) spectra in the spectral range of 4000–400 cm^−1^ were recorded with a JASCO FT/IR-4700 spectrometer (Jasco, Tokyo, Japan) equipped with a diamond crystal using a scanning speed of 128 scans/min, triangle apodization, and a resolution of 4 cm^−1^.

Raman spectra were recorded in extended mode using the 514 nm laser line, by measuring the Raman bands in the range of 100–3100 cm^−1^ monitoring shifts in the Raman band position narrower than 0.5 cm^−1^, using a high-resolution confocal Raman microscope (Renishaw system, from Renishaw Ltd., New Mills, Wotton-under-Edge Gloucestershire, UK) and a Leica DM2500 microscope (from Leica Microsystems GmbH, Wetzlar, Germany).

Scanning electron microscopy (SEM) analysis was performed on a Hitachi SU8230 (Hitachi, Tokyo, Japan) microscope at an acceleration voltage of 30 kV. The secondary electrons signal was registered. Before being analyzed, all samples were covered with a 9-nm-thick layer of gold, using a Quorum Q150T ES turbomolecular pumped coater (Quorum Technologies, London, UK).

Textural analysis of the samples was performed through N_2_ physisorption at −196 °C using a Micromeritics ASAP 2020 analyzer (Norcross, GA, USA). Before each measurement, the samples were degassed under vacuum at 120 °C for 12 h. The specific surface areas were calculated using the Brunauer–Emmett–Teller (BET) equation and the total pore volume was estimated from the amount adsorbed at the relative pressure of 0.99. The Barrett–Joyner–Halenda (BJH) model was used to determine the pore size distribution (PSD) curves from the adsorption data.

### 2.3. Catalytic Tests

Catalytic tests for the oxidation of IC in simulated wastewater were performed in a batch system under stirring at 150 rpm at 25 °C using 1 wt.% catalyst (particle size 0.16–0.25 mm) and H_2_O_2_ (30 wt.%) as an oxidation agent at different molar ratios H_2_O_2_/IC in the range 32–64. Three simulated wastewater samples having concentrations of IC in the range of 15 × 10^−3^ to 90 × 10^−3^ M were prepared. Blank tests (without catalyst in the reaction mixtures) were performed for each reaction condition.

Five recycling tests were performed only for the most active catalyst (HTMo-20GO). Thus, the catalyst recovered after the first reaction cycle (2 h, 150 rpm, 25 °C, 1 wt.% catalyst, 30 × 10^−3^ M initial concentration of IC and 48/1 molar ratio H_2_O_2_/IC) was used in the following reaction cycle using a fresh sample of simulated wastewater.

The IC concentration before and after the catalytic test was determined by UV-Vis spectroscopy considering the intensity of the absorption maximum at λ = 610 nm and a calibration curve obtained on a Jasco V-650 double-beam UV-Vis spectrometer (Jasco, Tokyo, Japan). The conversion of IC was calculated with the formula:

(2) $$IC\;conversion=\left[\frac{\left(IC_{0}-IC_{t}\right)}{IC_{0}}\right]\cdot 100\left(\%\right)$$

where *IC*_o_ is the molar concentration of *IC* at the beginning of the test, and *IC_t_* is the remaining *IC* concentration at the end of the test.

The concentrations of H_2_O_2_ at the beginning and end of the catalytic tests were determined by the spectrophotometric methods 209 and 210 developed on an Aqualytic spectrophotometer AL 800/SpectroDirect (Aqualytic Gmbh, Dortmund, Germany) using the specific reagent kits for the concentrations range of 0.01–0.5 mg/L and 0.03–1.5 mg/L H_2_O_2_.

The chemical oxygen demand (COD) and the total organic carbon (TOC) content in the treated wastewater were two other analysis methods used for the assessment of IC degradation. Both COD and TOC were determined using the Aqualytic AL800 spectrometer and the corresponding reagent kits COD Vario tube tests 0–1500 mg/L and 0–150 mg/L from Tintometer GmbH, Division Aqualytic (Dortmund, Germany) and TOC Cell test (50–800 mg/L) from Merck KgaA (Darmstadt, Germany).

## 3. Results

### 3.1. Characterization of the Materials

The obtained solids were characterized using ICP-AES, determination of acid and base sites, powder XRD, ATR-FTIR, Raman spectroscopy, scanning electron microscopy (SEM), and N_2_ adsorption-desorption isotherms.

#### 3.1.1. Chemical Composition and Acid-Base Properties

The results of the ICP-AES analysis, displayed in Table 1, indicate that as the amount of graphene oxide increases, both Mg/Al and Mo/Al atomic ratios decrease, implying that the presence of GO during synthesis results in an incomplete precipitation of Mg and Mo. The loss of Mo is higher than that of Mg most probably because at least a part of the GO platelets occupy some of the anionic exchange positions in the interlayer region of the LDH where Mo is also accommodated as molybdate anions. This trend is more intense as the content of GO in the synthesis increases.

The pyridine (Py) adsorption tests showed that all the hybrid samples had fewer acid sites than GO, but more than HTMo (Table 2). It should also be noted that the proportion of strong base sites (SB) determined by phenol adsorption decreases with the increase in GO content incorporated in the solids whereas the proportion of Brønsted acid sites (HB) increases. The sample HTMo-20GO exhibited the highest overall basicity and a Mo/Al ratio that is almost half that of the one used in the synthesis mixture.

#### 3.1.2. Powder XRD Characterization

**Figure 2:**
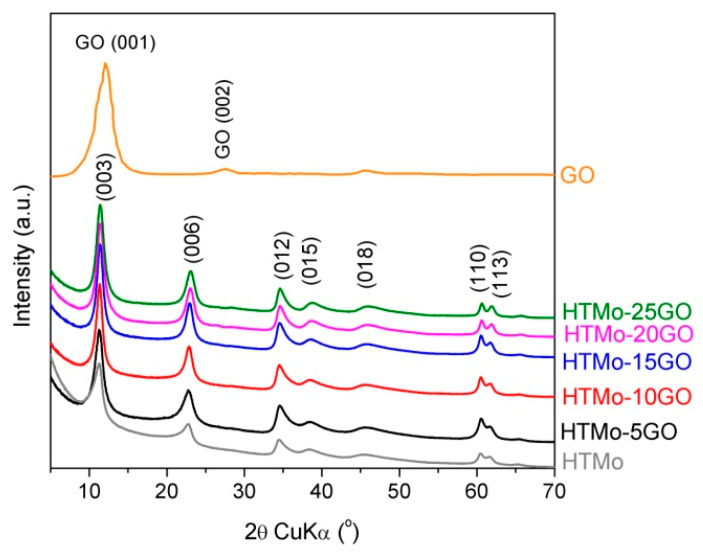
XRD patterns of HTMo-xGO materials compared to HTMo and GO.

The XRD patterns of the HTMo-xGO composites show the formation of single-phase layered double hydroxides without any impurities and confirm that the GO content introduced during synthesis has an influence on the intensity of the diffraction lines. Practically, the insertion of large amounts of graphene oxide during the synthesis of the materials led to higher-intensity lines, as can be observed in Figure 2. The absence of the intense GO (001) reflection indicated the exfoliation of the graphene sheets [24].

The lattice parameters and the crystallite sizes are given in Table 3. The crystallite sizes were calculated along two directions: perpendicular (D_003_) and parallel (D_110_) to the brucite-like layers, respectively. Due to the layered morphology of LDH, for the Scherrer mean crystallite sizes, the widths of two reflections were used: (003), related to the c-axis along which the layers are stacked, and (110), related exclusively to the brucite-like sheets. Table 3 shows the similar structural characteristics of all the samples. The *a*-lattice parameter corresponds to the distance between two metals cations in the brucite-like sheet and depends only on Mg/Al molar ratio, while the *c*-lattice parameter depends on both the Mg/Al ratio and the size of the interlayer anions [2]. The lower Mg/Al molar ratios obtained for the composite sample in comparison with the nominal Mg/Al = 3 obtained for a reference LDH Mo-free Mg_3_Al-CO_3_^2−^ prepared under the same conditions [33,34] are consistent with the evolution of the lattice parameters as revealed in Figure S1 in the Supplementary Materials. The reference samples for the Mg_x_Al-CO_3_^2−^ (x = 3 and 2.5, labeled HT3-CO_3_ and HT2.5, respectively) were extracted from our previous works [33,34,56]. As we had already asserted, the *c* lattice parameters, and hence the interlayer spaces, are large enough to accommodate MoO_4_ species [33].

The XRD patterns of the samples exhibit a better overall crystallinity compared to the molybdate ion embedded LDH prepared at a lower Mg/Al value [35] and, moreover, as mentioned, the GO presence, in particular for 10–25 wt.% range concentration, improved the samples crystallinities. From the data in Table 3, it can also be noticed that the presence of GO affected mostly the crystallite sizes, in particular the coherence lengths in the layer-stacking direction (D_003_).

The fact that larger LDH particles were formed after the insertion of GO suggested that GO sheets could serve as nucleating agents for LDH phase formation [57]. For the HTMo-xGO series, the evolution of the absolute intensities of the (110) line, exclusively related to the brucite-type layer, should go along with the decrease in the proportion of the LDH phase in the nanocomposites. In fact, the data in Table 3 show a decrease in the *c*-lattice parameter value accompanied by an increase in the I_003_/I_110_ ratio for all the HTMo-*x*GO nanocomposites compared to HTMo sample. This fact indicates a slight modification of the interlayer anionic composition due to mutual electrostatic interactions between the LDH and GO phases. It may be inferred that there is probably a higher degree of hydration with a different compaction of the anionic species.

#### 3.1.3. Characterization by Infrared Spectroscopy

**Figure 3:**
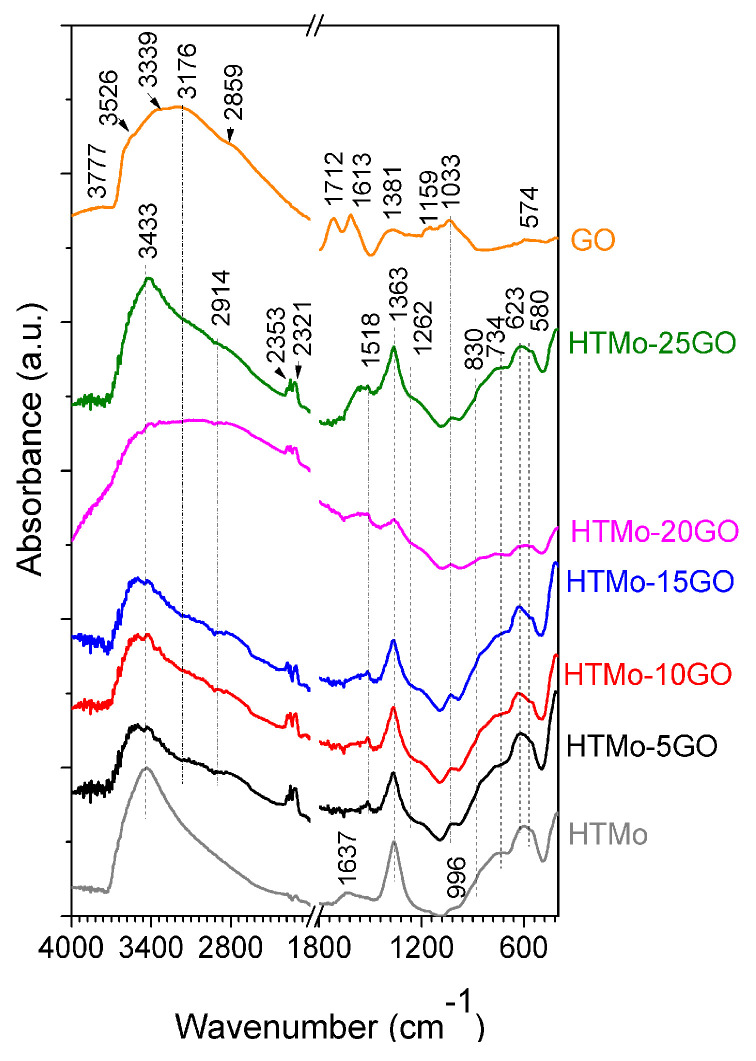
ATR-FTIR spectra of the hybrid catalysts, compared to neat HTMo and GO.

ATR-FTIR spectra of the hybrid solids are presented in Figure 3. The spectrum of HTMo has the main absorption bands at 3433 cm^−1^ (characteristic for OH stretching vibrations), 1637 cm^−1^ (corresponding to interlayer water bending modes), 1363 cm^−1^ (indicating the presence of carbonate ions in a symmetric environment [58,59]), 996 cm^−1^ (attributed to the antisymmetric mode of Mo–O–Mo characteristic for molybdate anions (MoO_4_^2−^) [35,59]), 830 cm^−1^ (stretching vibration in MoO_4_^2−^ [60]), and 734 cm^−1^, 623 cm^−1^, and 580 cm^−1^ (specific to the vibrations mode of the oxygen atoms bonded to Mg and Al from the crystal lattice [35]). Considering that Na_2_CO_3_ was not utilized in the synthesis, the presence of carbonate was probably due to the carbonation of the NaOH used in the preparation. The spectrum of neat GO shows the characteristic band for carboxylic groups present on the GO surface at 1712 cm^−1^ and a sharp peak at 1613 cm^−1^ associated with the stretching and bending vibration of OH groups in water molecules adsorbed on the GO. A broad band in the region 3600–2500 cm^−1^ (with inflections at 3526, 3339, and 2859 cm^−1^) appeared due to the stretching vibrations of OH groups. Other bands were observed too, such as a weak band at 3777 cm^−1^ corresponding to phenolic OH groups, a band at 1381 cm^−1^ due to C–OH bond vibrations, a band at 1033 cm^−1^ (vibration mode of aromatic C–O bonds), a band at 1159 cm^−1^ (corresponding to skeletal deformations), and a low-intensity peak at around 548 cm^−1^ due to C–H vibrations in the aromatic ring [61]. As can be seen, the bands corresponding to the neat HTMo and GO overlap across the entire spectral domain. In the spectra of all hybrid HTMo-xGO samples, a doublet appears at 2353 and 2321 cm^−1^ indicating CO_2_ entrapment in the solids. Other bands common to all the hybrids and not discernable in the neat HTMo and GO spectra are those at 1519 cm^−1^ and 1262 cm^−1^, which indicate the perturbance of the interlayer region of the LDH due to the incorporation of GO. The intensity of the bands corresponding to the vibrations mode of the oxygen atoms bonded to Mg and Al from the crystal lattice decreases as the concentration of GO increases. However, it is noticeable that the decrease in the case of HTMo-20GO is more accentuated than that for HTMo-25GO. Compared to HTMo, the relative intensity of the bands in the region 4000–2800 cm^−1^ decreases with the increase in the concentration of GO included in the hybrids up to 15 wt.%. Meanwhile, for the samples with 20 and 25% GO, the relative intensity of the bands in this region is higher than that for HTMo. For HTMo-20GO this band is visibly broader, a fact that explains its higher basicity (see Table 2). The band corresponding to Mo–O–Mo vibrations, which appeared at 996 cm^−1^ in the spectrum of HTMo, is overlapped by the band at 1033 cm^−1^ characteristic to GO in the spectra of the HTMo-xGO hybrids. Moreover, it can be observed that this band has a lower intensity for the samples having higher GO concentration suggesting that the amount of molybdate anions decreases with the increase in graphene oxide concentration, as could also be seen in the ICP-AES results (Table 1).

#### 3.1.4. Characterization by Raman Spectroscopy

**Figure 4:**
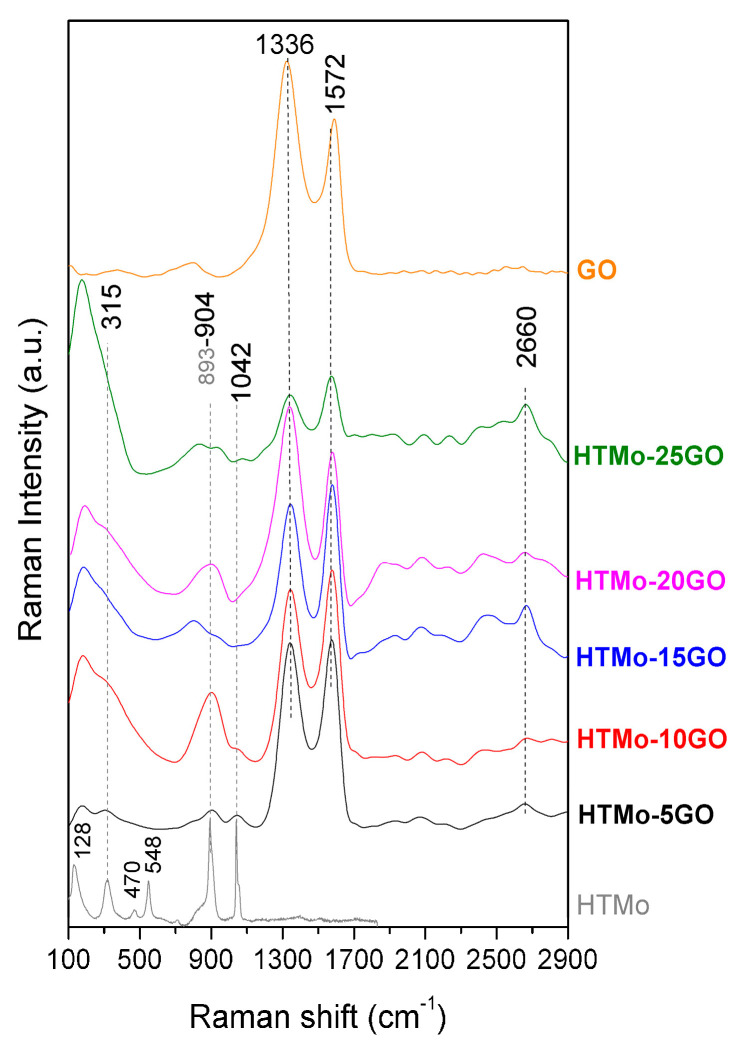
Raman spectra of the hybrid catalysts compared to neat HTMo and GO.

In the Raman spectra presented in Figure 4, for HTMo, the most intense band is the one appearing at 893 cm^−1^ which can be attributed to the Mo–O symmetrical stretching vibration in MoO_4_^2−^ (Mo in tetrahedral coordination), while the band at 315 cm^−1^ can be associated to the Mo=O bending vibrations [33]. The second most intense band appears at 1042 cm^−1^. This could indicate either contamination of the sample with carbonate since the ν_1_ symmetric stretch of A1′ symmetry of carbonate anion is known to be positioned at 1030 cm^−1^ [58] or the presence of Mo in octahedral coordination. The absorption bands present at 470 and 548 cm^−1^ are specific for the bending vibrations of Mg–OH, Al–OH.

In the Raman spectra of the hybrid materials, the signal coming from the graphene oxide component masks the bands emitted by the neat HTMo in the region 100–1100 cm^−1^, even though the concentration of the HTMo was higher than the GO concentration. However the spectra of the composites containing GO reveal the presence of a Mo–O symmetrical stretching vibration in MoO_4_^2−^ based on the absorption band at 904 cm^−1^ which is shifted from 893 cm^−1^ due to the insertion of graphene oxide. The band at 1042 cm^−1^ present in the spectrum of the HTMo is clearly visible only for the composite sample containing 5% GO. For all HTMo-*x*GO composites, the presence of GO was noticed in the Raman spectra displayed in Figure 3. In the spectra, the presence of GO is signified by the presence of the D band, (the dominant sp^2^ Raman signature of disorder in nanocrystalline carbonic structures at 1336 cm^−1^) and the G band at 1572 cm^−1^ (related to planar carbonic structures with sp^2^ hybridized C atoms) [62]. For the neat GO, the ratio of the intensities of the two bands I_D_/I_G_ = 1.43. In the spectra of the hybrid materials, the G’ band appears at around 2600 cm^−1^ and it increases in intensity with increasing graphene oxide content inserted in the composites. This fact suggests that as the GO concentration increases, some GO remains dispersed on the surface of the LDH particles, leading to an agglomeration of intertwined layers, as was noticed in the SEM micrographs.

#### 3.1.5. Characterization Using SEM Microscopy

**Figure 5:**
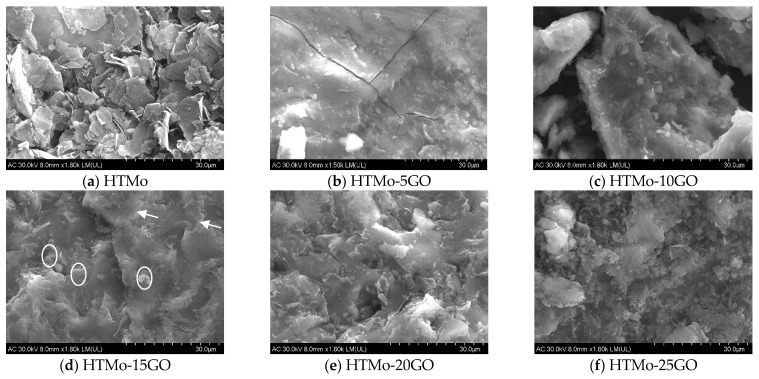
SEM analysis of the hybrid materials compared to HTMo: (**a**) HTMo; (**b**) HTMo-5GO; (**c**) HTMo-10GO; (**d**) HTMo-15GO; (**e**) HTMo-20GO; (**f**) HTMo-25GO.

SEM images of HTMo and HTMo-xGO samples containing 5–25 wt.% GO are displayed in Figure 5. HTMo exhibited the typical sheet-like morphology of LDH. The samples with 5–25 wt.% GO had a distinctive morphology compared to HTMo. After adding various concentrations of GO, the layered aggregates became more compact, and smaller particles can be noticed on their surface. The sample HTMo-5GO presented small particles dispersed on a thin layer, while, when increasing the amount of graphene oxide from 10 to 25 wt.%, a mixture of smaller particles dispersed on the surface of the layered aggregates can be noticed. These changes in morphology could be caused by the deposition of isolated GO particles on the external surface of the LDH layers [31] as inferred from the Raman analysis results.

#### 3.1.6. Textural Characterization

The textural features of the samples, as revealed by nitrogen adsorption–desorption, are displayed in Table 4. The adsorption–desorption isotherms for all samples (Supplementary Materials Figure S2) can be classified as type IV with a combination of H2a and H2b hysteresis loops associated with inkbottle-shaped pores resulting in significant network effects [63].

The small surface area of the neat HTMo may be a consequence of the sticking and twisting of the sheets as was observed by SEM analysis (Figure 5a). The samples HTMo-15GO and HTMo-25GO had larger specific surface areas. For HTMo-15GO, this fact may be a consequence of the pleated edges of the layers indicated by white arrows and the dispersion of small grains (indicated by white circles) on their surface, as revealed by SEM analysis (Figure 5d), whereas for HTM-25GO, there are numerous small grains dispersed between larger agglomerates (Figure 5f). The fact that HTMo-20GO has a lower specific surface area may be a consequence of the larger dimension of the particles (Figure 5e) compared to those noticed on HTMo-15GO and HTMo-25GO. These solids also have a bimodal pore size distribution (Supplementary Materials Figure S3). Meanwhile, the neat GO shows an H4-type hysteresis loop associated with narrow slit-like pores, including some microporosity [24], and has a monomodal type pore size distribution. HTMo-20GO exhibited a higher amount of wider pores (10.9 nm) than all the other hybrid samples.

### 3.2. Catalytic Tests Results

The results of the catalytic tests for indigo carmine oxidation with H_2_O_2_ after 2 h at room temperature and 150 rpm using different molar ratios H_2_O_2_/IC and 1%wt. catalyst are displayed in Table 5. It was found that the conversion of H_2_O_2_ is greater than that of indigo carmine dye (IC), as a result of not only the oxidation of IC but also the decomposition of a small amount of H_2_O_2_. The tests on H_2_O_2_ decomposition using the same amount of catalyst, in the absence of IC, revealed that after 2 h, the level of H_2_O_2_ decomposition was in the range of 4–6%, while without the catalysts, it was less than 3%. Under similar conditions the conversions of IC and H_2_O_2_ both increase with the GO loading in the hybrid catalysts up to 20% GO. The conversion is slightly lower for the sample with GO content of 25% than for the one with 20% GO. The increase in the ratio H_2_O_2_/IC in the reaction mixture leads to enhanced IC conversions for the catalysts HTMo, HTMo-5GO, HTMo-10GO, and HTMo-15GO, while it has a lower influence for the catalysts HTMo-20GO and HTMo-25GO. For all catalysts, the conversion of H_2_O_2_ was lower as the ratio H_2_O_2_/IC increased beyond the stoichiometric value of 32 by 25 up to 100%.

The increase in the initial concentration of IC in the range of 15 × 10^−3^ M to 90 × 10^−3^ M when using a molar ratio H_2_O_2_/IC of 48 in the reaction mixture led to a decrease in IC conversion (Table 6) by 7–10% for all catalysts except HTMo-20GO. This catalyst also had the highest overall basicity (Table 2) and the widest pores (e.g., 10.9 nm—Table 4) among the hybrid catalysts. The COD and TOC were measured for the highest IC concentration of 90 × 10^−3^ M. The results, shown in Supplementary Materials Table S2, indicate that COD was less than 200 mgO_2_/L for all catalysts, and TOC was below the detection limit for all hybrid catalysts and 55 mgC/L for neat HTMo. These COD values comply with Romanian regulations for the discharge of treated water into natural receptors (maximum 300 mgO_2_/L [64]).

## 4. Discussion

**Figure 6:**
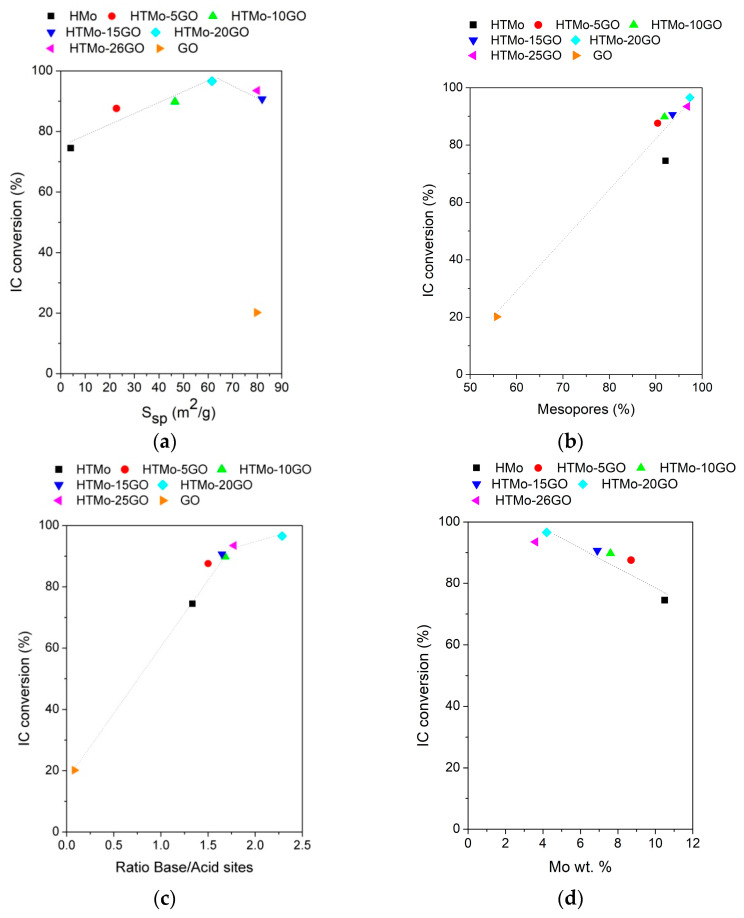
Dependence of IC conversion on the physico-chemical characteristics of the investigated catalysts: (**a**) specific surface areas; (**b**) the proportion of mesopores; (**c**) the basicity expressed as the ratio between base and acid sites; (**d**) Mo concentration (wt.%); (Reaction conditions: IC_0_ = 30 × 10^−3^ M, H_2_O_2_/IC = 48 catalysts concentration 1 wt.%, 150 rpm, 2 h, 25 °C).

For the Mo-containing catalysts, the conversion of IC increased with the specific surface area up to a maximum value of 96.6 % at S_sp_ 61.6 m^2^/g (catalyst HTMo-20GO—Figure 6a). The conversion of IC rises with the proportion of mesopores in the catalysts due to the easier accessibility of the IC to the catalytically active sites. The increase fitted a linear trend for all the samples containing GO, while HTMo was a little below this trend, probably due to its much lower surface area (Figure 6b).

There is a linear increase in IC conversion with the basicity of the catalysts up to a value of 1.73 of the ratio of base/acid sites (corresponding to HTMo-25GO) followed by a slight increase beyond this value (Figure 6c). This fact may be related to the increase in the single molecular oxygen generation from hydrogen peroxide in contact with the base sites of the Mo-LDH phase [32,38]. The results showed that the conversion of indigo carmine dye (IC) varied according to the Mo concentration, as shown in Figure 6d. The variations were similar for all the investigated H_2_O_2_/IC ratios. For the hybrid catalysts, the conversion decreases linearly with the increase in Mo amount, suggesting that at lower concentrations, there are larger spaces between Mo active sites on the surface thus avoiding their screening by the large molecules of IC.

The conversion of H_2_O_2_ was found to be less influenced by the surface area of the catalysts, with the hybrid composites displaying a higher conversion than the single-phase samples, HTMo and GO (Supplementary Materials Figure S4a).

The number of mesopores (Supplementary Materials Figure S4b) and the basicity (Figure S4c) show the same influence on H_2_O_2_ conversion as in the case of IC conversion. The presence of Mo leads to an increased conversion of H_2_O_2_ compared to that obtained on GO. The results indicate that the utilization of a hybrid catalyst, which combines the properties of Mo-modified layered double hydroxides (HTMo) and graphene oxide (GO), results in increased H_2_O_2_ conversion compared to using either HTMo or GO alone. This is because the hybrid catalyst provides both Mo sites and oxygen-containing functional groups from GO, whereas using GO alone only offers the latter. The hybrid catalysts exhibit a 15–16% increase in H_2_O_2_ conversion compared to HTMo and a 50–51% increase compared to GO, as demonstrated by the data in Supplementary Materials Figure S4a–c under the specified reaction conditions. H_2_O_2_ conversion varied depending on the amount of Mo reaching a maximum value for the HTMo-15GO sample (Supplementary Materials Figure S4d). The relationship between IC and H_2_O_2_ conversion, considering factors like surface area, mesopore proportion, basicity, and Mo concentration is consistent for all H_2_O_2_/IC ratios.

**Figure 7:**
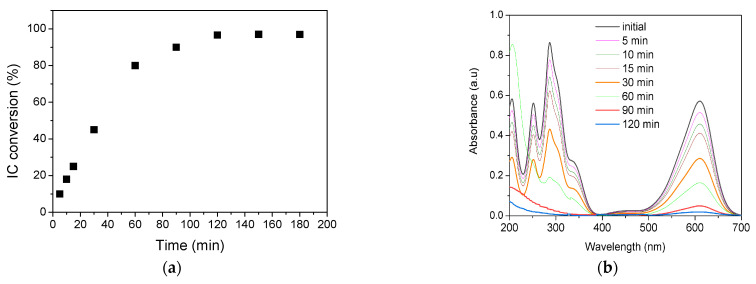
IC conversion on HTMo-20GO (Reaction conditions: IC_0_ 30 × 10^−3^ M, H_2_O_2_/IC = 48 catalysts concentration 1 wt.%, 150 rpm, 2 h, 25 °C); (**a**) Temporal variation; (**b**) UV-Vis spectra of the initial wastewater and during the process.

For the most promising catalyst, HTMo-20GO, the variation in IC conversion was determined during 180 min, analyzing the water samples after 5, 10, 15, 30, 60, 90, 120, 150, and 180 min of reaction time. The results, plotted in Figure 7a, show a rapid increase in the conversion up to 80% in the first 60 min, and a slower increase up to 120 min, at which point a plateau is reached. The UV-Vis spectra collected at the beginning of the test and during the process are shown in Figure 7b. The spectra obtained after 150 and 180 min are not presented due to their overlapping with the spectrum obtained after 120 min. Considering the modifications noted in the spectra during the process, it may be inferred that the oxidative degradation starts after an induction period of 30 min since all the absorption bands characteristic for IC are still present in the spectra. At 60 min reaction time, there is a notable increase in the charge transfer band located at 210 nm while the absorption maximum at 251 nm disappears and the maximum at 287 nm decreases significantly. After 90 min, both absorption maxima at 287 nm and 251 nm characteristic of aromatic intermediates [50] are lost, indicating the quasi-total mineralization of IC.

**Figure 8:**
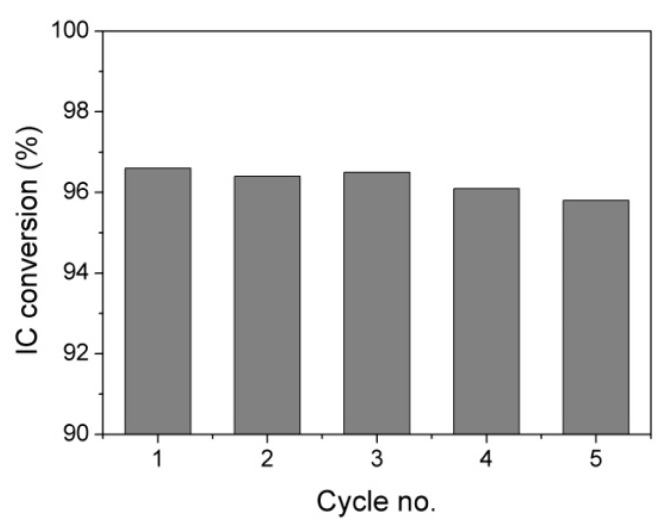
IC conversion in five reaction cycles on HTMo-20GO (Reaction conditions: IC initial concentration 30 × 10^−3^ M, H_2_O_2_/IC = 48 catalysts concentration 1 wt.%, 150 rpm, 2 h, 25 °C).

**Figure 9:**
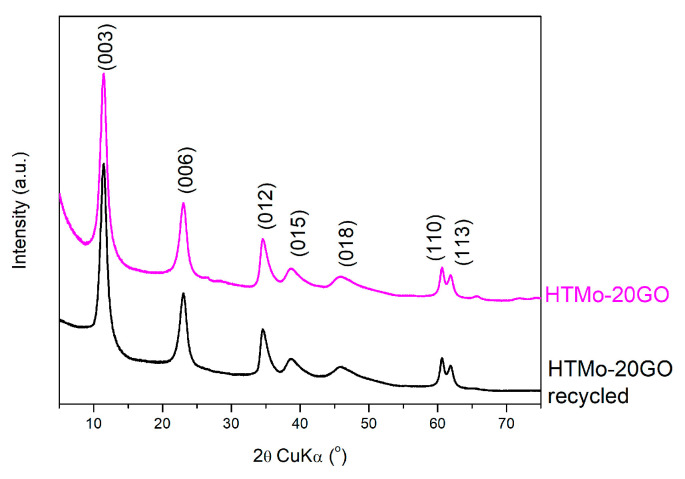
XRD patterns of HTMo-20GO before and after five reaction cycles.

The results of the recyclability tests performed on HTMo-20GO (Figure 8) show that the catalyst is stable for at least five reaction cycles since the conversion of IC decreases by less than 1% which is within the limit of experimental errors. The XRD pattern of the HTMo-20GO catalyst recovered after the fifth cycle (Figure 9) does not show alterations compared to the pattern of the fresh HTMo-GO indicating the stability of the catalyst.

Compared to photocatalytic IC degradation with TiO_2_ photocatalyst [50], our catalysts present the advantage of enabling the removal of IC without leaving colorless organic compounds in amounts exceeding the allowed levels of COD in the treated wastewater (see Supplementary Materials Table S2) and without requiring a UV source for the activation. There is also no need to perform photocatalytic degradation at temperatures higher than 25 °C (40 °C) and acidic pH (e.g., 2–4) in order to reach a high degradation rate of the dye [51,52]. Our catalyst is also more active than MnO_2_ catalyst [53] enabling 95.4% vs. 70% degradation of IC at similar initial concentrations of IC (e.g., 42 mg/L (90 × 10^−3^ M) for HTMo-20GO and 40 mg/L for MnO_2_) at lower catalyst loadings (1 wt.% HTMo-20GO < 1.4 wt.% MnO_2_) without requiring the use of ultrasonication equipment. In terms of stability, the HTMo-20GO catalyst was more stable than a Cu-hematite-based nanocatalyst, which lost 10% of its activity after the fifth reaction cycle under similar conditions [55]. For the other catalysts tested in the degradation of IC (TiO_2_, MnO_2_, Ti/IrO_2_-SnO_2_-Sb_2_O_5_ [50,51,52,53]) there were no reports related to their recyclability.

## 5. Conclusions

The co-precipitation of Mo-modified layered double hydroxides (Mo-LDH) in graphene oxide (GO) suspensions leads to the obtaining of single-phase hybrid materials without impurities. Compared to the composition of the synthesis mixture, the distribution of metal species in the hybrid materials reflected that the precipitation of the LDH-Mo was partially hindered by the presence of the GO suspension, and there was a significant loss of Mo with the increase in GO concentration. This fact could be a consequence of the competition between molybdate anions and GO-generated anions for the occupation of the interlayer space of the LDH. The insertion of GO enables the formation of larger LDH particles and a slight modification in the interlayer anionic composition. Hybrid materials have fewer acid sites than pure GO and more basic sites than GO and HTMo. On the hybrid catalysts, the activation of H_2_O_2_ was enhanced by an additive effect of molybdate sites and GO. The conversion of indigo carmine (IC) over the prepared hybrid catalysts increases with their specific surface area and basicity, reaching a maximum value of 96.6% when HTMo-20GO was used.

Mo-LDH-GO materials are environmentally friendly, easily recoverable, and reusable, making them a promising solution for treating dye-contaminated wastewater by advanced oxidation processes. Further experiments will be devoted to the study of the influence of salt-type additives present in IC-contaminated wastewater.

## Figures and Tables

**Table 1:** Metal content in the samples as determined by ICP-AES analysis.

Catalysts	Metal Content (wt.%)			Atomic Ratios	
	Mg^2+^	Al^3+^	Mo	Mg/Al	Mo/Al
HTMo	15.9	6.1	10.5	2.90	0.48
HTMo-5GO	14.5	5.7	8.7	2.83	0.43
HTMo-10GO	13.6	5.4	7.6	2.80	0.40
HTMo-15GO	12.6	5.0	6.9	2.80	0.39
HTMo-20GO	11.3	4.6	4.2	2.73	0.26
HTMo-25GO	10.0	4.1	3.6	2.71	0.25

**Table 2:** Distribution of acid and base sites in the samples.

Catalysts	Total Acid Sites (mmol Py/g)	HB ^1^	Total Base Sites (mmol AA/g)	SB ^2^	Base/Acid Sites Ratio
		(%)		(%)	
HTMo	0.15	10.1	0.20	25.1	1.33
HTMo-5GO	0.18	14.2	0.28	20.2	1.55
HTMo-10GO	0.25	18.4	0.40	17.4	1.60
HTMo-15GO	0.34	24.6	0.48	14.7	1.65
HTMo-20GO	0.20	21.3	0.52	10.8	2.40
HTMo-25GO	0.30	27.5	0.56	8.2	1.73
GO [24]	0.77	31.2	0.06	0	0.08

^1^—HB-Brønsted acid sites determined by pyridine adsorption from the areas of the corresponding peaks in IR spectra as reported in reference [24]. ^2^—SB-Strong base sites determined by phenol adsorption.

**Table 3:** Structural data for HTMo and HTMo-xGO hybrids.

Samples	Lattice Parameters		Crystallite Sizes		I_003_/I_110_	I_110(HTMO)_/ I_110(HTMo-xGO)_
	*a* (Å)	*c* (Å)	D_003_ (nm)	D_110_ (nm)		
HTMo	3.061	23.814	12.7	6.8	4.4	1.00
HTMo-5GO	3.058	23.582	10.4	6.5	4.2	0.85
HTMo-10GO	3.059	23.509	12.4	8.3	5.2	0.84
HTMo-15GO	3.058	23.326	12.3	8.3	4.7	0.99
HTMo-20GO	3.055	23.232	13.7	7.2	6.4	0.94
HTMo-25GO	3.054	23.271	15.0	6.9	6.7	0.92

**Table 4:** Results of the textural analysis using nitrogen adsorption–desorption analysis.

Samples	S_sp BET_ (m^2^/g)	t-Plot Micropore Area (m^2^/g)	t-Plot External Surface Area (m^2^/g)	BJH Adsorption Average Pore Width (nm)	BJH Adsorption Cumulative Volume of Pores (cm^3^/g)	Pore Size ^1^ nm
HTMo	4.1	0.3	3.8	17.5	0.020	2.7 and 18.1
HTMo-5GO	22.7	2.0	20.8	8.6	0.057	2.7 and 9.1
HTMo-10GO	46.5	3.5	43.0	8.5	0.116	2.4 and 9.0
HTMo-15GO	82.9	5.0	77.9	8.6	0.209	2.7 and 9.0
HTMo-20GO	61.6	1.6	60.0	10.2	0.185	2.4 and 10.9
HTMo-25GO	80.9	2.5	78.4	8.0	1.197	2.6 and 9.0
GO [24]	79.8	24.5	55.3	5.5	0.059	3.9

^1^ From Supplementary Materials Figure S3.

**Table 5:** The conversion of IC and H_2_O_2_ using HTMo-xGO catalysts at different molar ratios H_2_O_2_/IC (initial concentration of IC 30 × 10^−3^ M, catalysts concentration 1 wt.%, 150 rpm, 2 h, 25 °C).

Catalysts	Molar Ratios H_2_O_2_/IC									
	32.1		40		48		56		64	
	IC Conv. (%)	H_2_O_2_ Conv. (%)	IC Conv. (%)	H_2_O_2_ Conv. (%)	IC Conv. (%)	H_2_O_2_ Conv. (%)	IC Conv. (%)	H_2_O_2_ Conv. (%)	IC Conv. (%)	H_2_O_2_ Conv. (%)
HTMo	66.1	70.1	71.3	61.2	74.5	53.8	79.1	49.3	82.8	45.5
HTMo-5GO	82.7	86.7	83.7	71.2	87.6	62.6	89.6	55.4	90.4	49.3
HTMo-10GO	83.4	87.4	87.6	74.1	89.8	64.1	91.4	56.4	91.8	50.0
HTMo-15GO	86.1	98.6	90.3	80.1	90.7	68.6	91.8	58.9	92.3	51.1
HTMo-20GO	94.6	95.4	94.8	78.1	96.6	66.5	95.7	57.5	95.9	52.2
HTMo-25GO	91.4	90.1	92.3	76.5	93.5	64.7	93.4	56.6	93.7	53.0
GO	12.3	16.3	16.2	17.0	20.2	17.5	22.1	16.7	23.5	15.8
blank	1.5	5.5	3.1	6.5	4.2	6.8	5.3	7.0	6.4	7.2

**Table 6:** The influence of the initial concentration of IC on the catalytic activity of HTMo-xGO catalysts at molar ratio H_2_O_2_/IC = 48 (catalysts concentration 1 wt.%, 150 rpm, 2 h, 25 °C).

Catalysts	IC_o_ = 15 × 10^−3^ M		IC_o_ = 90 × 10^−3^ M	
	IC Conv. (%)	H_2_O_2_ Conv. (%)	IC Conv. (%)	H_2_O_2_ Conv. (%)
HTMo	78.4	56.4	68.7	55.7
HTMo-5GO	90.1	64.3	81.2	64.9
HTMo-10GO	91.4	65.1	84.7	67.5
HTMo-15GO	92.2	65.7	85.4	68.1
HTMo-20GO	98.2	69.7	95.4	75.6
HTMo-25GO	96.4	68.5	88.3	70.2
GO	35.4	27.7	26.5	24.1
blank	4.6	7.1	3.5	6.8

## Data Availability

The data presented in this study are available upon request from the corresponding author.

## Supplementary Materials

The following supporting information can be downloaded at: https://www.mdpi.com/article/10.3390/ma16083025/s1, paragraph S1: The preparation of the GO suspension; Figure S1: Variation in the lattice parameters determined by XRD depending on the chemical composition of the brucite-type layer: (a) variation in *a* parameter, (b) variation in *c* parameter; Figure S2: BET isotherms of the investigated samples; Figure S3: Pore size distribution of the investigated samples (BJH, Halsey–Faas correction); Figure S4: The influence of the physico-chemical characteristics of the investigated catalysts on H_2_O_2_ conversion: (a) specific surface areas; (b) the proportion of mesopores; (c) the basicity expressed as the ratio between base and acid sites; (d) Mo concentration; (Reaction conditions: IC_0_ = 30 × 10^−3^ M, H_2_O_2_/IC = 48 catalysts concentration 1 wt.%, 150 rpm, 2 h, 25 °C); Table S1: Compositions of the solutions A and B used in the syntheses of HTMo-xGO hybrids; Table S2: The chemical oxygen demand (COD) and total organic carbon content (TOC) of the water samples after the catalytic tests performed with a concentrated solution of IC (0.09 mM; COD initial 574.7 mg O_2_/L; TOC initial—174.2 mg C/L) at a molar ratio H_2_O_2_/IC = 48/1.
