# Supplementary material for: Mo-LDH-GO Hybrid Catalysts for Indigo Carmine Advanced Oxidation
Source: Materials (Basel). 2023 Apr 11;16(8):3025. doi: 10.3390/ma16083025 (PMC10142217; doi:10.3390/ma16083025)
Supplement: Supplementary file 1 [file materials-16-03025-s001.zip › materials-2253989-s001.pdf]

materials

MDPI

Article

Mo-LDH-GO Hybrid Catalysts for Indigo Carmine Advanced Oxidation

Octavian Dumitru Pavel[1,2], Alexandra-Elisabeta Stamate[1,2], Rodica Zăvoianu[1,2,*], Anca Cruceanu[1,2], Alina Tirsoaga[2], Ruxandra Bîrjega[3], Ioana Andreea Brezeștean[4], Alexandra Ciorîță[4,5], Daniela Cristina Culiță[6] and Ana Paula Vieira Soares Dias[7]

[1] University of Bucharest, Faculty of Chemistry, Department of Inorganic Chemistry, Organic Chemistry, Biochemistry and Catalysis, 4-12 Regina Elisabeta Bd., 030018 Bucharest, Romania
[2] Research Center for Catalysts & Catalytic Processes, Faculty of Chemistry, University of Bucharest, 4-12 Regina Elisabeta Bd., 030018 Bucharest, Romania
[3] National Institute for Lasers, Plasma and Radiation Physics, 409 Atomistilor Street, PO Box MG-16, 077125 Măgurele, Romania
[4] National Institute for Research and Development of Isotopic and Molecular Technologies, 67-103 Donat Street, 400293 Cluj-Napoca, Romania
[5] Electron Microscopy Centre, Faculty of Biology and Geology, Babes-Bolyai University, 44 Republicii Street, 400015 Cluj-Napoca, Romania
[6] Ilie Murgulescu Institute of Physical Chemistry, 202 Splaiul Independentei, 060021 Bucharest, Romania
[7] CERENA, Instituto Superior Técnico, Universidade de Lisboa, 1 Rovisco Pais Av., 1049-001 Lisboa, Portugal
* Correspondence: rodica.zavoianu@chimie.unibuc.ro

S1. The preparation of the GO suspension

The method applied for the preparation of graphene oxide is based on the technique developed by Hummers in 1952 [4, 24]. Thus, graphite powder (325 mesh, from Aldrich, Schnelldorf, Germany), sodium nitrate ($NaNO_3$) and potassium permanganate ($KMnO_4$, chemical purity, from Merck, Darmstadt, Germany), sulfuric acid (H_2SO_4, 98%, from Merck, Darmstadt, Germany), hydrochloric acid (HCl, 37%, from Merck), and hydrogen peroxide (H_2O_2, 30%, from Chimreactiv, Bucharest, Romania) were utilized. In brief, 23 mL concentrated sulfuric acid and 1 g of graphite were mixed in an Erlenmeyer flask maintained in an ice bath at 0 °C. Then, 0.5 g of sodium nitrate and 3.0 g of potassium permanganate were slowly added to the formed solution which was kept under continuous stirring for 30 min. Further, the paste was diluted with 46 mL of distilled water causing a strong exothermic effect accompanied by bubbling, and the resulting mixture was stirred for one hour at 90 °C. Moreover, to remove the residual permanganate, the whole solution was mixed with 71 mL of warm distilled water and 5 mL H_2O_2 and kept for one hour under stirring. For the removal of the remaining metal ions, 125 mL HCl 0.1 N was added, and the solution was stirred for another hour. The resulting mixture was centrifuged and washed with warm distilled water until the conductivity was below 100 μS/cm. The concentration of GO in the suspension was determined using the gravimetric method, by weighing 3 liquid samples of 100 mL in Petri dishes before and after the evaporation of water under vacuum at 60 °C for 24 h. The amount of solid recovered from the 3 samples was 0.4001, 0.4005, and 0.3998 g, which gave an average value of the GO concentration in the suspension of ca. 4 g/L.

Table S1. Compositions of the solutions A and B used in the syntheses of HTMo-xGO hybrids.

**Samples**	**Solution A**					**Solution B**		
	**Volume H_2O (mL)**	**Volume GO suspension (mL)**	**$Mg(NO_3)_2\cdot 6H_2O$ g/*mMoles***	**$Al(NO_3)_3\cdot 9H_2O$ g/*mMoles***	**GO (g)**	**Volume H_2O (mL)**	**NaOH g/*mMoles***	**$Na_2MoO_4\cdot 2H_2O$ g/*mMoles***
HTMo	847	0	22.72/*88.6*	11.08/*29.5*	0	845	10.4/*260*	3.6/*14.9*
HTMo-5GO	679.5	125	21.59/*84.2*	10.54/*28.1*	0.5	817.6	9.9/*247.5*	3.4/*14.1*
HTMo-10GO	519	250	20.49/*79.9*	10/*26.7*	1	782	9.4/*235*	3.24/*13.4*
HTMo-15GO	308.5	375	19.29/*75.3*	9.4/*25.1*	1.5	695	*8.84/221*	*3.05/12.6*
HTMo-20GO	150.6	500	18/*70.2*	8.79/*23.4*	2	661.6	8.24/*206*	2.84/*11.7*
HTMo-25GO	25	625	17/*66.3*	8.3/*22.1*	2.5	660	*7.8/195*	2.7/*11.2*

(a)

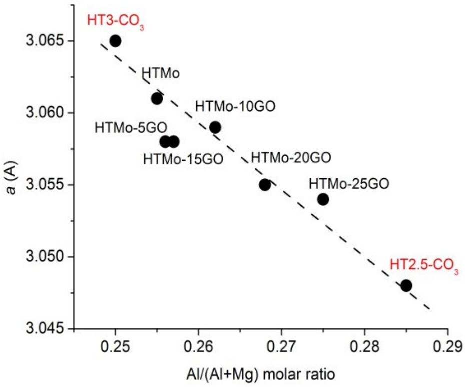

(b)

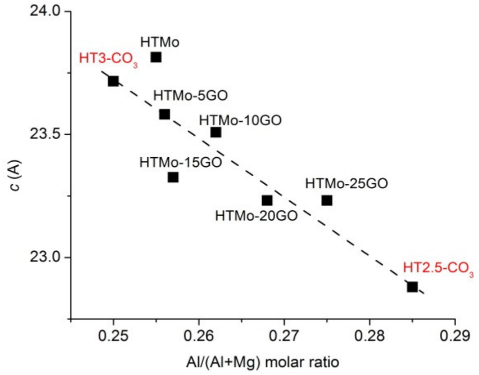

Figure S1. The linear variation of the lattice parameters determined by XRD depending on the chemical composition of the brucite type layer: (a) variation of *a* parameter, (b) variation of *c* parameter. The reference samples for the $Mg_xAl-CO_3^{2-}$ (x=3 and 2.5, labeled HT3-CO_3 and HT2.5, respectively) were extracted from our previous works [33, 34, 56].

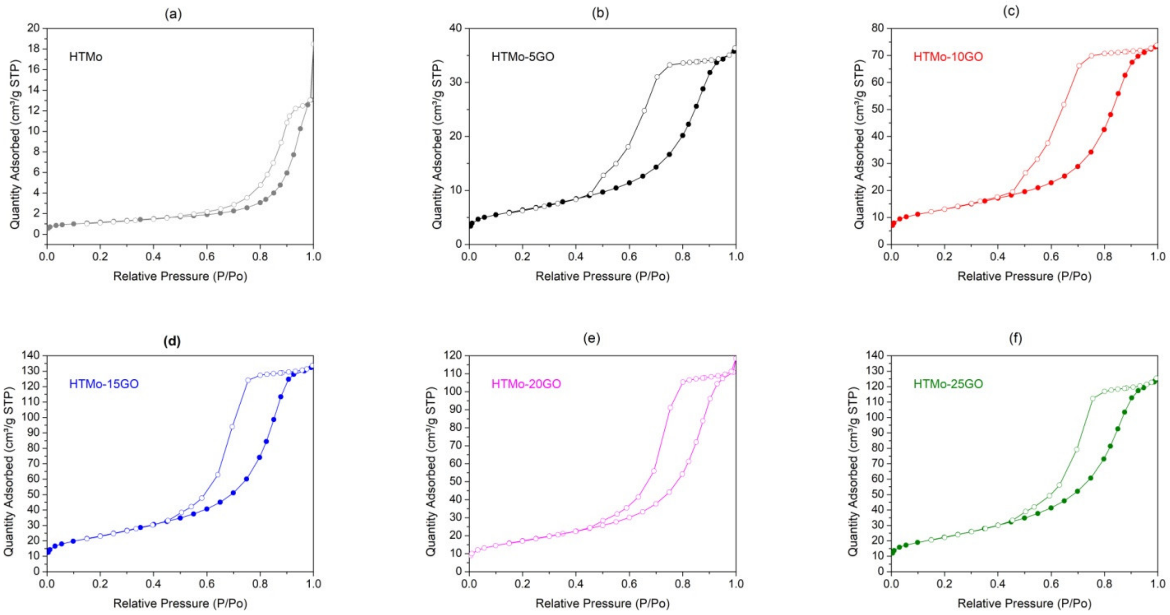

Figure S2. BET isotherms of the investigated samples.

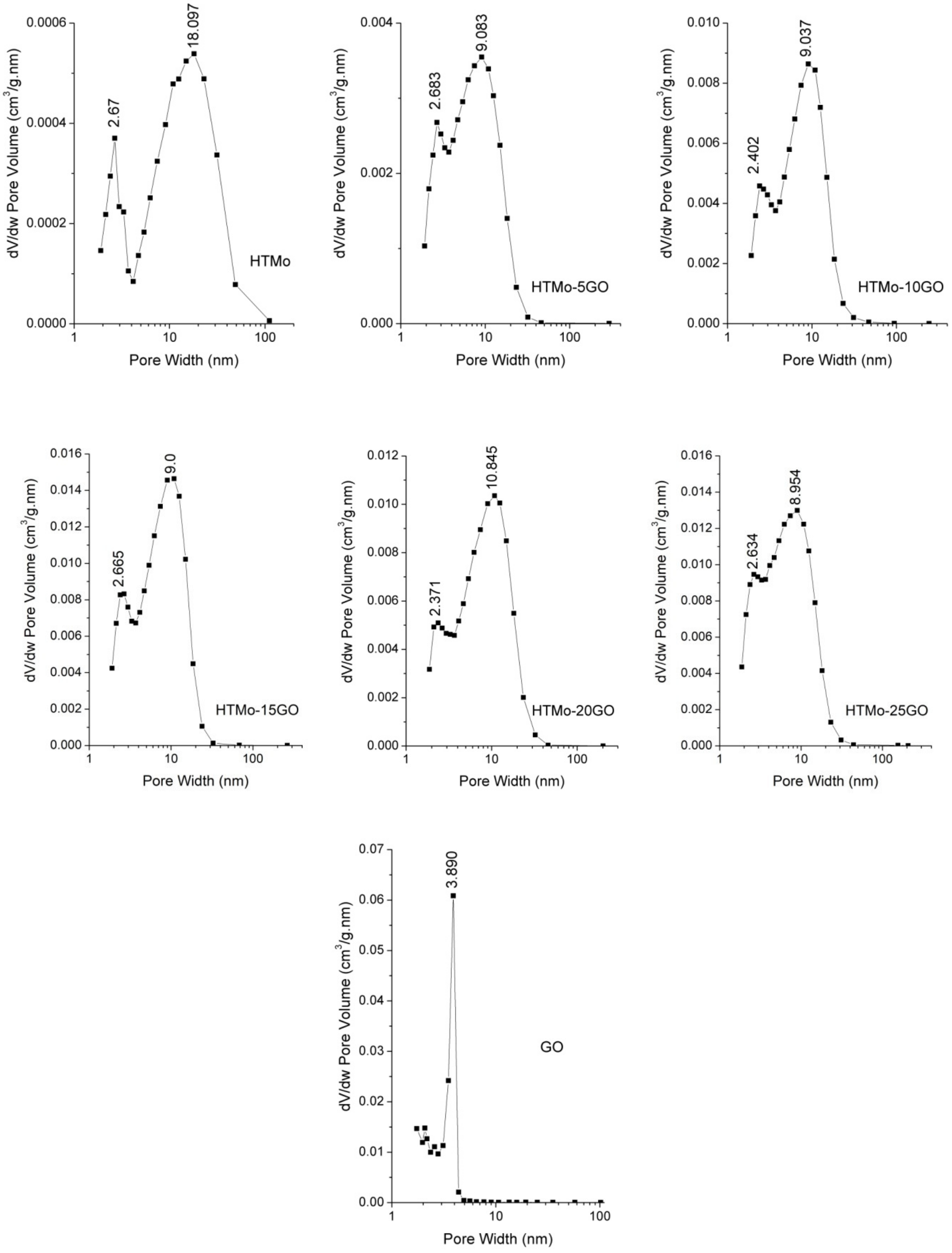

Figure S3. Pore size distribution of the investigated samples (BJH, Halsey-Faas correction).

Table S2. The chemical oxygen demand (COD) and total organic carbon content (TOC) of the water samples after the catalytic tests performed with concentrated solution of IC (IC_0=90x10^{-3} M; COD initial 574.7 mg O_2/L; TOC initial -174.2 mg C/L) at a molar ratio H_2O_2/IC=48/1.

Catalysts	IC conversion (%)	COD	TOC
HTMo	68.7	180	55
HTMo-5GO	81.2	108	u.d.l.
HTMo-10GO	84.7	88	u.d.l.
HTMo-15GO	85.4	84	u.d.l.
HTMo-20GO	95.4	27	u.d.l.
HTMo-25GO	88.3	67	u.d.l.

u.d.l. = under detection limit

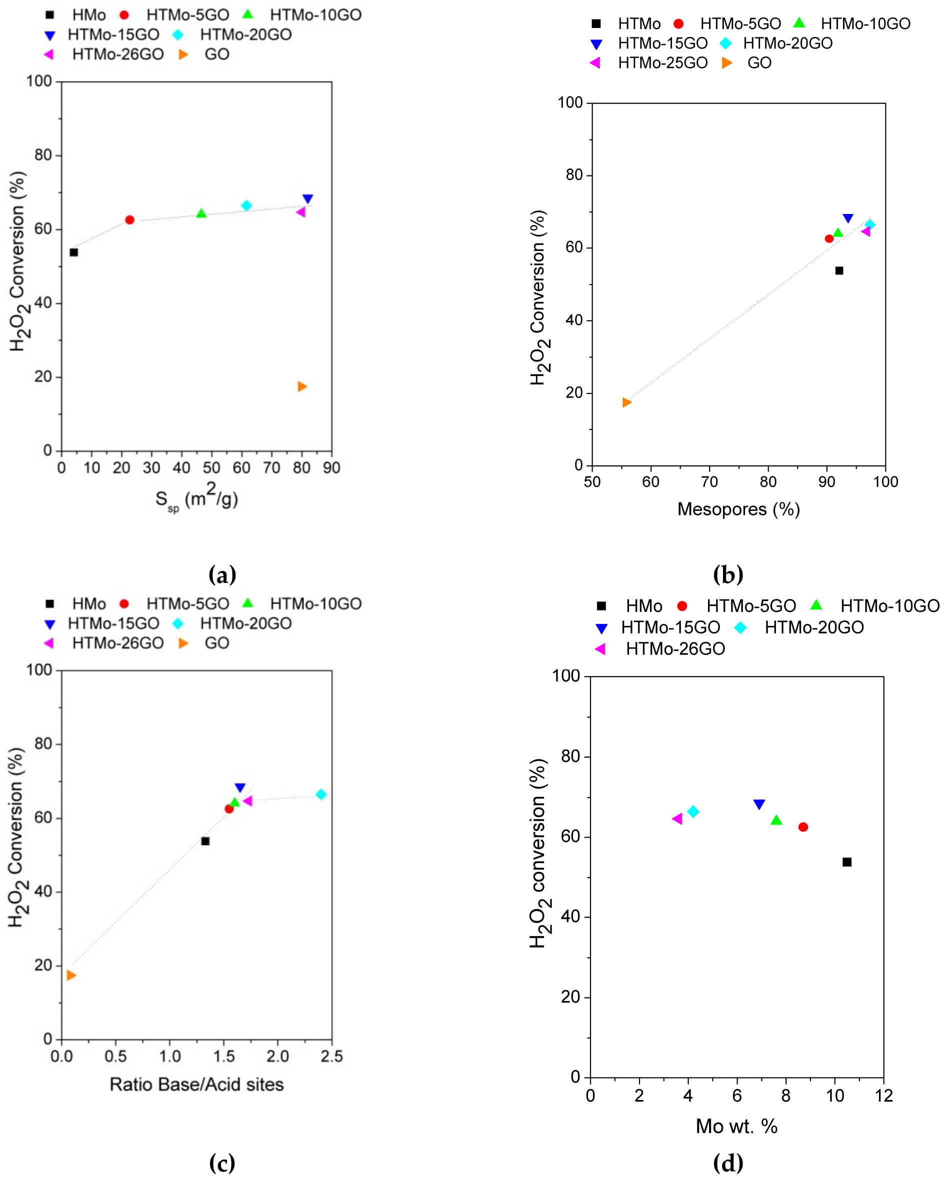

Figure S4. The influence of the physico-chemical characteristics of the investigated catalysts on H_2O_2 conversion: (a) specific surface areas; (b) the proportion of mesopores; (c) the basicity expressed as the ratio between base and acid sites; (d) Mo concentration; (Reaction conditions: IC_0=30x10^{-3} M, H_2O_2/IC=48 catalysts concentration 1 wt. %, 150 rpm, 2 h, 25 °C).

References

4. Hummers, W.S.; Offeman, R.E. Preparation of graphitic oxide. *J. Am. Chem. Soc.*, **1958**, *80*, 1339–1339. https://doi.org/10.1021/ja01539a017

24. Stamate, A.-E.; Pavel, O.D.; Zăvoianu, R.; Brezeştean, I.; Ciorîță, A.; Bîrjega, R.; Neubauer, K.; Koeckritz A.; Marcu, I.-C. Ce-containing MgAl-layered double hydroxide-graphene oxide hybrid materials as multifunctional catalysts for organic transformations. *Materials* **2021**, *14*, 7457. https://doi.org/10.3390/ma14237457

33. Zăvoianu, R.; Bîrjega, R.; Pavel, O.D.; Cruceanu, A.; Alifanti, M. Hydrotalcite like compounds with low Mo-loading active catalysts for selective oxidation of cyclohexene with hydrogen peroxide. *Appl. Catal. A: General* **2005**, *286, 211–220*. https://doi.org/10.1016/j.apcata.2005.03.009
34. Zăvoianu, R.; Cruceanu, A.; Pavel, O.D.; Angelescu, E.; Soares Dias, A.P.; Birjega, R. Oxidation of tert-butanethiol with air using Mo containing hydrotalcite-like compounds and their derived mixed oxides as catalysts. *React. Kinet. Mech. Catal.* **2012**, *105*, 145–162. https://doi.org/10.1007/s11144-011-0398-9
56. Zăvoianu, R., Pavel, O. D., Cruceanu, A., Florea, M., Bîrjega R. Functional layered double hydroxides and their catalytic activity 196 for 1,4-addition of n-octanol to 2-propenonitrile. *Appl. Clay. Sci.* **2017**, *146*, 411-422. https://doi.org/10.1016/j.clay.2017.06.030
